# Congenital Clubfoot: Difficulties in Maintaining the Use of the Dennis-Brown Orthosis by Caregivers

**DOI:** 10.1055/s-0044-1788785

**Published:** 2024-09-04

**Authors:** Fernando Minari Sassi, Filipe Augusto Shimanoe Nazario, Gabriela Bardelli, Ana Carolina Pauleto

**Affiliations:** 1 Faculdade Pequeno Príncipe, Curitiba, PR, Brasil; 2 FAE Centro Universitário, Curitiba, PR, Brasil

**Keywords:** clubfoot, foot deformities, foot orthoses, orthopedic devices

## Abstract

**Objective** This study aimed to identify the main difficulties faced by the family when a child with congenital clubfoot (CC) uses the Dennis-Brown orthosis.

**Method** This study interviewed via Google Forms caregivers of children treated from 2015 to 2018 regarding their difficulties in orthosis use.

**Results** The answers revealed that orthosis-related difficulties are independent of the child's gender, age, or affected side. We noted that 41.7% of the respondents reported some difficulty, especially the child's irritation when using the orthosis (93.3%).

**Conclusion** The main factor in CC relapses is poor adherence to orthosis use. As a result, studying factors causing or increasing the probability of interrupting orthosis use is significant in creating strategies to facilitate their use, potentially reducing CC recurrence.

## Introduction

Congenital clubfoot (CC) is a complex three-dimensional deformity including pes equinus, varus, adductus, and cavus. 1 CC results from ligamentous and myotendinous abnormalities and occurs in 1/1,000 live births. 2

The Ponseti method is the main form of treatment and consists of joint manipulations of the connective tissue using serial casts. After correction, the patient must use a Dennis-Brown (DB) orthosis for 2 to 4 years. 1

Many parents of children diagnosed with CC are afraid of the disease and its treatment. CC causes stress, anxiety, and depression in parents. 3 Therefore, it is critical to clarify all doubts regarding the therapeutic process. 4 The Ponseti method requires a lot of commitment from the medical team and the patient's family to be successful. 5 6

Considering the significance of full adherence to treatment, this study aimed to identify the main difficulties encountered in DB use by caregivers of CC patients.

## Method

This descriptive exploratory study relied on a quantitative approach to the difficulties of consistently using DB orthosis by children undergoing conservative CC treatment using the Ponseti method in a reference hospital in Curitiba, PR, Brazil, from 2015 to 2018. The Committee of Research Ethics from our institution approved the study under number CAAE 56086821.0.0000.5580. To collect the required information, we sent a 16-item questionnaire addressing the child, their caregivers, and difficulties in orthosis use to the caregivers. First, we contacted the caregivers (parents, grandparents, or others) by cell phone to inform them that they would receive a questionnaire via Google Forms and its purpose. The questionnaire could be answered by email or cell phone. This study included caregivers of children over 4 years old diagnosed with CC at birth and treated on an outpatient basis at the reference hospital.

## Statistical Analysis

We created an Excel spreadsheet with all the answers and inserted it into the Sphinx IQ2 software to analyze profile variables. Next, we proceeded to bivariate analyses to relate the data obtained. We performed three statistical tests (p-value, chi-square, and degree of freedom) concerning the variables looking for any statistical influence on the difficulty of using the orthosis.

## Results

Our sample consisted of 176 patients, and we managed to contact 114 of their caregivers. However, only 36 caregivers answered the form, with a 31.6% response rate. Most (88.9%) patients underwent treatment at the Brazilian Unified Health System (SUS, for its acronym in Portuguese), 8.3% received treatment through an insurance plan, and 2.8% had private treatment. The gender distribution revealed 63.9% of patients were males and 36.1% were female. In 55.6% of subjects, CC was bilateral. CC affected the right foot of 33.3% of patients and the left foot of 11.1% of subjects.

**Fig. 1:**
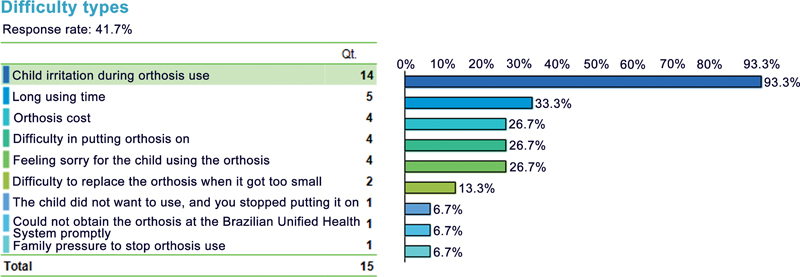
Types of difficulty faced by the patient. Source: Authors (2022).

There was no significant correlation between gender and the side affected by CC ( Fig. 1 ), meaning the reported statistical tests revealed no direct relationship between gender and deformity. Most (80.6%) children required a calcaneus tenotomy. Orthosis use after conservative treatment started when the child was less than 12 months old in 66.7% of patients and continued until 48 months old in 38.9% of children. Most (58.3%) caregivers denied any difficulty and 41.7% reported difficulties. There was no association between difficulty in orthosis use and CC side or patient's gender and age, demonstrating that difficulty is independent of these factors.

Of the 41.7% of caregivers reporting some orthosis-related difficulty, the main one was the child's irritation during its use (93.3%). Other issues highlighted included long using time (33.3%), orthosis cost (26.7%), difficulty in putting the orthosis on (26.7%), and feeling sorry for the child (26.7%). Caregivers also cited difficulty in changing the orthosis when it became too small (13.3%), the child not accepting its use (6.7%), failure to obtain orthosis from SUS promptly (6.7%), and pressure from family members to stop using the orthosis (6.7%). Discontinuation in orthosis treatment occurred in 27.8% of patients; of these, none reported CC recurrence but 30% reported the need to resume treatment.

## Discussion

The incidence of CC has had a similar pattern over the years: 64.5% in male patients and 35.5% in female patients in 1951, 2 and a predominance of male patients (68%) in 2020. 4 In our study, 63.9% of patients were male. Regarding the affected side, our sample showed a higher CC incidence on the right foot and a difference in frequency, with 75% affecting the right side and 25%, on the left side. In 2011, the incidence of right foot deformities was 56%. 7 In 2020, the same pattern remained, with a 52.1% incidence on the right foot. 4 As for bilateral (55.6%) and unilateral (44.4%) CC, our data are consistent with the literature. Stewart reported an equal distribution between bilaterality and unilaterality. 2

In our study, 66.7% of patients started CC treatment using the Ponseti method at less than 12 months old, and most (38.9%) completed it by 48 months old. This indicates that most patients underwent 4 years of treatment, consistent with the literature reporting an average follow-up period of 4.6 years. 6 Most (80.6%) of our patients required a percutaneous tenotomy of the calcaneus tendon, consistent with reports from Ponseti 8 and, more recently, Bhaskar and Patni. 9

In 2004, the lack of commitment to using the abduction orthosis was the main factor associated with CC recurrence, with these patients being 183 times more susceptible to relapses. 10 In 2011, researchers stated that the appropriate orthosis use directly relates to the treatment success. 11 A report from 2022 found that 46% of patients with relapses were incorrectly using the DB orthosis. 12 Of the 36 caregivers participating in our research, 10 (27.8%) reported having stopped using the orthosis before the stipulated treatment period. Among these ten caregivers, three (30%) had to restart treatment and resume orthosis use.

As mentioned before, almost 30% of our patients presented an increased risk of CC recurrence and potential surgical intervention. A report from 2015 identified that this inappropriate use increases the chance of needing a surgical procedure by 7.9-fold. 13 Furthermore, in 2013, researchers highlighted that treatment for recurrent CC is not simple and does not result in the same outcome of improving foot mobility and anatomy compared with conservative treatment. 14

A study from 2013 described that parents of patients who received greater focus on the need for adherence and clarification during classes about the disease had a lower recurrence and higher therapeutic success when compared with the group that did not receive these instructions 5 Similarly, in 2016, researchers stated the importance of commitment from the doctor and the patient's family from changing casts to orthosis use. 6 A report from 2011 identified some factors specific to parents that influence orthosis use, stating that parents with education levels of up to high school or less had a greater chance of their children developing relapses. 11

In our study, 41.7% of the sample presented some difficulty using the DB orthosis, consistent with a 2022 study on the Mitchell-Ponseti orthosis stating that 46.7% of families from CC patients reported issues with the orthosis. 15 Therefore, by integrating parents into the treatment, teaching them, and reinforcing the need for orthosis use, it may be possible to reduce orthosis use discontinuation or its irregular use since they would understand the direct relationship between success and proper use.

In 2022, authors identified two main factors for the decrease in compliance with orthosis use: first, after 18 months old, children sleep less, using the orthosis for less time, as they cannot tolerate it when awake. 16 The second point reported by these authors is that when faced with this difficulty, parents do not use the orthosis correctly or even discontinue its use. 16 Our research identified that the most important factor for irregular orthosis use is child irritation, reported by 93% of study participants, consistent with descriptions from previous authors.

Given the above, there is a need to address the reported factors to reduce CC recurrences. Sheta and El-Sayed, in 2020, did not use orthosis, teaching parents stretching exercises for daily performance, and giving them classes three times a week about the disease and its treatment. 4 The authors reported that the children of these parents had above-average results concerning functionality, pain, and other factors. However, the recurrence rate was 21%, consistent with the literature. 4 Therefore, despite removing the concern about inappropriate orthosis use, this method is not more efficient than the traditional one to the point of replacing orthosis completely.

Just as described in a 2022 study, the difficulties encountered by family members in using the orthosis did not influence therapeutic success. In our study, despite 27.8% having discontinued orthosis use, none reported CC relapse. 15 Therefore, although the literature states that inappropriate orthosis use is the main factor in CC recurrence, our study cannot state that orthosis-related difficulties are direct factors for its inappropriate use.

## Conclusion

Few studies specifically seek to understand why there are difficulties in using the DB orthosis, even though it is the main orthosis used in SUS. The 41.7% rate of DB orthosis use difficulty is unprecedented, and fundamental to creating strategies to reduce its inappropriate use. Furthermore, our data allows us to understand that despite the issues during orthosis use, they were not factors in CC recurrence. In addition, we noted that the difficulty is independent of the patient's gender, age, and affected side. However, the literature suggests that the socioeconomic condition of the parents impacts recurrence. Finally, the limitation of this study was the low questionnaire response rate.
