# Supplementary material for: Congenital Clubfoot: Difficulties in Maintaining the Use of the Dennis-Brown Orthosis by Caregivers
Source: Rev Bras Ortop (Sao Paulo). 2024 Sep 4;59(4):e613–6. doi: 10.1055/s-0044-1788785 (PMC11374405; doi:10.1055/s-0044-1788785)
Supplement: Related texts

# Research article: Pé torto congênito: Dificuldades na manutenção do uso da órtese de Dennis-Brown pelos cuidadores

**Keywords:** aparelhos ortopédicos, deformidades do pé, órteses do pé, pé torto equinovaro

## Abstract

**Objetivo I** dentificar as principais dificuldades apresentadas pela família durante o uso da órtese de Dennis-Brown.

**Método** Esse estudo foi realizado com os responsáveis por pacientes tratados no período de 2015–2018, os quais responderam a um formulário com perguntas sobre a criança por google forms quanto às dificuldades no uso da órtese.

**Resultado** Com as respostas obtidas, identificamos que as dificuldades com a órtese independem do sexo, idade ou lado afetado na criança. Foi possível identificar que 41,7% dos pacientes que fizeram o uso da órtese apresentaram algum tipo de dificuldade, sendo a principal dificuldade a irritação da criança (93,3%).

**Conclusão** Sabendo que o principal fator de recidivas do PTC é a má aderência ao uso das órteses, o estudo de fatores que causam ou aumentam a probabilidade da interrupção do seu uso se torna um importante aliado para a criação de estratégias para facilitar o uso da órtese, assim possivelmente diminuindo a recidiva da doença.

## Introdução

O pé torto congênito (PTC) é uma deformidade complexa tridimensional, como pé equino, varo, aduto e cavo, 1 associado a alterações ligamentares e miotendinosas, observada em 1/1000 nascidos vivos. 2

O método de Ponseti é a principal forma de tratamento e consiste em manipulações articulares do tecido conectivo através da utilização de gessos seriados. Após correção, é indicado o uso de órtese Dennis-Brown (DB) por um período de 2 a 4 anos. 1

Muitos pais de crianças com diagnóstico de PTC ficam receosos com a doença e com seu tratamento, podendo ser um fator de estresse, ansiedade e depressão para eles, 3 sendo assim, é de suma importância a elucidação de todas as dúvidas referentes ao processo terapêutico. 4 O tratamento pelo método de Ponseti exige muito comprometimento tanto da equipe médica quanto da família do paciente para se alcançar o sucesso. 5 6

Tendo em vista a importância da adesão plena ao tratamento, o estudo visa identificar as principais dificuldades encontradas na utilização da órtese de DB pelos cuidadores de pacientes com PTC.

## Método

Estudo exploratório descritivo com abordagem quantitativa relacionado às dificuldades na manutenção do uso da órtese de DB em crianças que realizaram o tratamento conservador de PTC pelo método de Ponseti em um hospital de referência na cidade de Curitiba durante o período de 2015 a 2018, aprovado pelo Comitê de Ética em Pesquisa da nossa instituição sob o número CAAE: 56086821.0.0000.5580. As informações foram coletadas através do preenchimento de questionário composto por 16 perguntas, as quais abordaram dados sobre a criança, cuidadores e as dificuldades no uso da órtese. Os cuidadores (pai, mãe, avós ou outros) foram contatados inicialmente por celular para tomarem ciência de que receberiam um questionário pela plataforma em questão ( *Google Forms* ) e qual era a finalidade deste, podendo ser respondido por e-mail ou pelo celular. Foram incluídos os cuidadores de crianças maiores de 4 anos com diagnóstico de PTC ao nascimento tratadas ambulatorialmente no hospital de referência.

## Análise Estatística

Uma planilha em Excel com todas as respostas obtidas no formulário foi criada e inserida dentro do software Sphinx IQ2. Dessa forma, foram criadas análises de variáveis de perfil e após isso análises bivariadas buscando relacionar os dados obtidos. Foram realizados 3 testes estatísticos (p-value, qui-quadrado e grau de liberdade) na relação das variáveis onde procurávamos alguma influência estatística sobre a dificuldade no uso da órtese.

## Resultados

Dos 176 pacientes, foi possível o contato de 114 cuidadores, sendo que apenas 36 responderam ao formulário com porcentagem de adesão de 31,6%. Desses pacientes, 88,9% realizaram o tratamento pelo SUS, enquanto que 8,3% realizaram pelo convênio e 2,8% de forma particular. A distribuição por sexo foi de 63,9% masculino e 36,1% feminino. Em relação ao lado mais acometido, 55,6% eram bilaterais, 33,3% à direita e 11,1% à esquerda.

Ao correlacionar sexo e o lado mais acometido afetado pelo PTC ( Fig. 1 ), não houve nenhuma relação significativa, ou seja, não encontramos uma relação direta do sexo com a deformidade (avaliado pelos testes estatísticos relatados). A indicação da tenotomia do tendão de Aquiles foi necessária em 80,6% dos pacientes. O início do uso da órtese pós-tratamento conservador em 66,7% dos pacientes iniciou com menos de 12 meses de vida e a manutenção do uso foi até os 48 meses (38,9%). Relatos quanto à dificuldade ocorreram em 41,7% dos pacientes, enquanto 58,3% negaram e não foi observado dificuldade relacionada ao lado acometido pelo PTC, ao sexo e à idade, demonstrando que a dificuldade independe destes fatores.

**Fig. 1:** Tipos de dificuldade apresentada pelo paciente. Fonte: Autores (2022).

Dos 41,7% dos pacientes que tiveram algum tipo de dificuldade no uso da órtese, a principal delas foi a irritação da criança no uso desta (93,3%). Outros problemas apontados com destaque foram o longo tempo de uso (33,3%), custo da órtese (26,7%), dificuldade na colocação do aparelho (26,7%) e sentimento de pena da criança (26,7%). Também foram citados: dificuldade de troca do aparelho quando este ficava pequeno (13,3%), criança não aceitava o uso (6,7%), não obtenção da órtese pelo SUS no tempo adequado (6,7%) e pressão de familiares para parar de usar o aparelho (6,7%). A interrupção no tratamento com uso da órtese ocorreu em 27,8% dos pacientes, dos quais nenhum relatou recidiva da deformidade, mas 30% relataram a necessidade de voltar a utilizar a órtese.

## Discussão

A incidência do PTC possui um padrão similar ao longo dos anos: 64,5% em pacientes masculinos e 35,5% nos femininos no ano de 1951, 2 enquanto que em 2020, ainda se encontra uma predominância de 68% masculinos. 4 Nesta pesquisa, 63,9% dos pacientes também eram masculinos. Quanto ao lado acometido, em nossa amostra, encontrou-se uma maior incidência de deformidade no pé direito e houve diferença na frequência, sendo de 75% direito em relação a 25% esquerdo. Em 2011, identificou-se uma incidência de 56% para as deformidades do pé direito. 7 Ainda em 2020, o mesmo padrão se manteve, com uma incidência de 52,1% do pé direito. 4 Quando comparado a bilateral (55,6%) e unilateral (44,4%), nossos dados se assemelham a literatura: Stewart relatou uma distribuição igual entre a bilateralidade e unilateralidade. 2

Em nosso estudo, 66,7% dos pacientes iniciaram o tratamento do PTC pelo método de Ponseti com menos de 12 meses de idade, enquanto que a maioria (38,9%) finalizou até os 48 meses de idade, o que indica que a maioria dos pacientes realizaram os 4 anos de tratamento concordando com o observado na literatura, uma média de 4,6 anos de seguimento. 6 Quanto à tenotomia percutânea do tendão de Aquiles, foi necessária em 80,6% dos pacientes participantes desse estudo conforme com o descrito na literatura por Ponseti 8 ; e, mais recentemente, Bhaskar e Patni. 9

Em 2004, foi identificada a falta de comprometimento no uso da órtese de abdução como o principal fator associado à recidivas, sendo estes pacientes 183 vezes mais suscetíveis a recidiva da deformidade. 10 Em 2011, pesquisadores afirmaram que o uso adequado da órtese possui relação direta com o sucesso do tratamento. 11 Em 2022, apontou-se que 46% dos pacientes que apresentaram recidivas faziam o uso incorreto da órtese de DB. 12 Dos 36 responsáveis que participaram da nossa pesquisa, 10 (27,8%) relataram ter interrompido o uso da órtese antes do período estipulado de tratamento. Destes 10, 3 (30%) tiveram que reiniciar o tratamento e retomada do uso da órtese.

Tendo em vista o parágrafo anterior, foi identificado que quase 30% dos pacientes de nossa pesquisa apresentaram riscos aumentados de recidiva da deformidade, assim como para uma possível intervenção cirúrgica. Em 2015, foi identificado que esse uso inadequado aumenta em 7,9 vezes a chance da necessidade de um procedimento cirúrgico. 13 Além disso, pesquisadores já em 2013 destacaram que o tratamento em pés recidivados não é simples e não possui o mesmo resultado na melhora da mobilidade e anatomia do pé que o tratamento conservador. 14

Em 2013, foi descrito que pais dos pacientes que receberam maior enfoque na necessidade de adesão e esclarecimentos durante aulas sobre a doença tiveram menor recidiva e maior sucesso terapêutico, quando comparados ao grupo que não teve o mesmo. 5 Assim como no estudo comentado, em 2016, pesquisadores afirmaram a importância do compromisso tanto do médico quanto da família do paciente, desde o período de troca de gessos até o do uso da órtese. 6 Em 2011, foi identificado alguns fatores próprios dos pais que influenciam o uso da órtese, relatando que pais com níveis de educação de até o ensino médio ou menos tinham maiores chances de seus filhos desenvolverem a recidiva. 11

Neste estudo, 41,7% da amostra apresentou algum tipo de dificuldade durante o uso da órtese de DB, assemelhando-se com um estudo de 2022, sobre a órtese de Mitchell-Ponseti, que identificaram que 46,7% das famílias de pacientes com PTC relataram dificuldades com a órtese. 15 Portanto, ao integrar os pais ao tratamento, ensinando-os e reforçando a necessidade da órtese, é possível que ocorra a diminuição da interrupção do uso do equipamento, ou seu uso irregular, já que é possível entender que eles possuem uma relação direta com o uso adequado.

Já em 2022, autores identificaram 2 fatores principais para a diminuição da conformidade do uso do aparelho: primeiro, após um ano e meio de idade, as crianças tornam a dormir menos, usando por menos tempo, já que não o toleram quando acordadas. 16 O segundo ponto que os autores relatam é que, diante dessa dificuldade, os pais não fazem o uso correto ou até abandonam completamente o seu uso. 16 Através da nossa pesquisa, identificamos que em nossa amostra, o fator mais importante para o uso irregular da órtese é a irritação da criança, visto em 93% dos participantes do estudo, assim assemelhando-se ao descrito pelos autores anteriores.

Diante do exposto, existe a necessidade de enfrentar os fatores relatados para assim diminuir as recidivas do PTC. No estudo de Sheta e El-Sayed, 2020, os autores não utilizaram órteses, ensinando aos pais exercícios de alongamento para serem realizados diariamente, além de 3 dias de aula por semana sobre a doença e tratamento. 4 Os autores relatam que os pacientes tiveram resultados acima da média em relação a funcionalidade, dor e outros fatores, porém tiveram 21% de recorrência das deformidades, dentro do que é encontrado na literatura. 4 Portanto, apesar de retirar a preocupação com o uso inadequado da órtese, o método encontrado não é mais eficiente que o tradicional a ponto de substituir o uso da órtese completamente.

Assim como descrito em um estudo de 2022, que as dificuldades encontradas pelos familiares no uso da órtese não influenciaram o sucesso terapêutico, em nosso estudo, apesar de 27,8% ter interrompido o uso da órtese, nenhum destes relatou retorno da deformidade. 15 Portanto, apesar da literatura afirmar que o uso inadequado da órtese é o principal fator de recidiva do PTC, em nosso estudo não foi possível afirmar que as dificuldades no uso dela são fatores diretos para o uso inadequado.

## Conclusão

Existem poucos estudos que procuram entender especificamente o porquê das dificuldades no uso da órtese de DB, mesmo ela sendo a principal órtese utilizada no SUS. O resultado de 41,7% de dificuldade no uso da órtese de DB além de inédito, é um dado importante para ser utilizado como base para a criação de estratégias na redução do uso inadequado do aparelho. Além disso, os dados encontrados nessa pesquisa permitem o entendimento que apesar da presença das dificuldades com o uso da órtese, elas não foram fatores para recidiva da doença. Também, foi identificado que a dificuldade é um fator independente do sexo, idade e lado afetado do paciente, porém existe a sugestão da literatura de que ela seja afetada pela condição socioeconômica dos pais. Por fim, a limitação deste estudo foi a baixa adesão ao formulário de respostas.
